# Effects of nitrogen fertilization on global xylem transcript profiling of *Eucalyptus urophylla x grandis* evaluated by RNA-seq technology

**DOI:** 10.1186/1753-6561-5-S7-P106

**Published:** 2011-09-13

**Authors:** Eduardo Camargo, Leandro Costa, Marçal Soler, Marcela Salazar, Jorge Lepikson, Danieli Gonçalves, Wesley Marques, Marcelo Carazzolle, Yves Martinez, Jacqueline Grima-Pettenati, Gonçalo Pereira

**Affiliations:** 1 Laboratório de Genômica e Expressão, IB, UNICAMP, Campinas, São Paulo, Brazil; 2 Génomique Fonctionnelle de l’Eucalyptus, LRSV, UPS/CNRS, Castanet-Tolosan, France; 3 Federative Research 3450, Castanet-Tolosan, France

## Background

Eucalyptus species are the most widely planted hardwood trees in the world representing more than 4.75 million ha in Brazil. Their high productivity, valuable wood properties and wide adaptability could allow sustainable and cost-efficient production of lignocellulosic bioenergy. The main limitation to this objective is wood recalcitrance to degradation which is linked to the structure and composition of lignified secondary cell walls. Lignin, for example, impairs the accessibility of cellulose during kraft pulping as well as during saccharification, a key step of bioethanol production.

The application of nitrogen fertilizers is one strategy to increase growth rates and productivity since nitrogen is one of the most limiting nutrient for tree growth and carbon sequestration. However, the effects of nitrogen availability on wood properties and related gene expression are poorly understood.

In poplar, it was recently reported that N fertilization increased aerial biomass, while in wood, fibre morphology and secondary cell wall structure and composition were modified. An increase in cellulose coupled with a decrease in lignin was observed and the mRNA profiles evaluated by microarray showed that nitrogen and tension wood have overlapping effects [1]. Moreover, a highly significant genetic correlation was observed between plant growth and lignin/cellulose composition. Quantitative trait loci co-localization identified the genomic position of potential pleiotropic regulators [2].

In order to get an insight on the regulation of nitrogen availability on wood formation in Eucalyptus, we have studied the effects of nitrogen fertilization on xylem transcriptome profiles using RNA-seq technology.

## Methods

An experimental system was set up in which rooted cuttings of *Eucalyptus urophylla* x *grandis* were fertilized during 30 days with three different amounts of N (limiting, -N; adequate, CT; excess, +N). For the treatment with excess of N fertilization, we used two different nutrient solutions with different concentration of NO_3_^-^ and NH_4_^+^.

Histochemical analyses were performed on stem transverse sections (80 μm thick) obtained using a Vibratome (LEICA VT 1000S). The Weisner reagent (phloroglucinol- HCl) was used in order to detect lignified cell walls, and calcofluor reagent to evaluate the cellulose content by fluorescence. The samples were observed in confocal microscopy (SP2-AOBS, Leica) and under bright-field microscopy (DM IRBE, Leica) coupled with a CCD camera (DFC 300 FX, Leica).

The construction of the RNA-seq libraries and sequencing were performed accordingly to Illumina’s protocols. Prior to analyze the RNA-seq data, we have done an assembly of Eucalyptus ESTs (GENOLYPTUS and NCBI). The 53,412 unigenes produced were automatically annotated using BLAST (e-value cutoff of 1e-5) against different sequence databases.

The RNA-seq reads were aligned against the assembled unigenes using the SOAP2 aligner [3] configured to allow up two mismatches, discard sequences with “N”s and return all optimal alignments. In order to perform the differential expression analysis between libraries, a normalization and statistics pipeline were applied using DEG-seq software [4] considering 99% of confidence rate (cut-off value of 0.01).

## Results and discussion

The intensity of staining with phloroglucinol was higher in stem sections from samples grown under -N treatment as compared to control, whereas it was lower in both +N treatments. This suggests that lignin biosynthesis is decreased in presence of excess of nitrogen fertilizer. We could also observe in the +N samples, an increase in cellulose staining intensity with the calcofluor reagent specially noticeable when comparing with -N samples suggesting that nitrogen also influence cellulose biosynthesis.

The RNA-seq analysis generated 123,121,154 sequence reads after filtering. Of the total reads, about 86 millions matched either to a unique (47,9%) or to multiple (21,8%) EST locations. Each nitrogen treatment was represented by at least 28.8 million reads, a tag density sufficient for quantitative analysis of gene expression.

The sequence reads were aligned on the new *Eucalyptus* EST assembly resulting in 36,125 unigenes expressed (15,293 contigs and 20,832 singlets). After statistics analysis, we determined 14,400 differentially expressed genes, that for a preliminary analysis were divided in two scenarios: the genes down-regulated in -N and up-regulated in both +N treatments (8,967 genes), and the genes up-regulated in –N and down-regulated in both +N treatments (5,433 genes).

To facilitate the global analysis for each scenario, gene ontology (GO) classification (http://www.geneontology.org) was performed and showed differences in some biological process categories. For instance, we observed that genes involved in the biosynthesis of cell wall main components (lignin, cellulose and hemicelluloses) were differentially expressed between the treatments. For example, some genes of the lignin biosynthetic pathway were up-regulated in the treatment with less nitrogen (-N) and down-regulated in both +N treatments.

## Conclusions

This first analysis in Eucalyptus allowed us to show an effect of nitrogen fertilization on cell wall composition both at the histological and at the gene expression level. We believe that further experimentscompleted by chemical analysis of wood samples will give more insights in the mechanisms of nitrogen fertilization on wood formation. This knowledge will be important to address new demands on better suited biomass quality for industrial applications.
